# The scientific position of Qualitative studies-comprehensive understanding of health and well-being

**DOI:** 10.1080/17482631.2019.1667661

**Published:** 2019-10-09

**Authors:** Henrika Jormfeldt

**Affiliations:** Department of Health and Welfare, Halmstad University, Halmstad, Sweden

Qualitative studies give emphasis to the perspective of individuals and their experiences, meanings, situations and actions through the eyes of those who are familiar with these experiences. Qualitative research has been referred to as being an act of both art and science (Holloway & Todres, 2007), thus the purpose of qualitative research is not just a scientific concern with truth but also an aesthetic and ethical matter of meaning. Deegan (1996) has described the difference between pure cognitive knowledge as recognition of the already approved facts unlike wisdom which involves a deeper understanding of the essence of the already known content, through metaphors of the human heart. The human heart is described as; the heart that can break; the hardened heart; the cold heart; the heart that aches; the heart that stands still; the heart that leaps with joy; and the one who has lost heart in contrast to the knowledge about the objectively described pump that beats in any person (Deegan, 1996).

Similarly, health could be described from several different perspectives. Objectively, health has shown to be positively related to concepts such as self-esteem, empowerment and quality of life, but to a minor extent negatively associated to symptoms of illness, discrimination and rejection experiences (Jormfeldt, Arvidsson, Svensson, & Hansson, 2008). Accordingly, subjective goals and desires of the individual are vital to health and well-being and to the extended understanding of the subjective experiences of contents. The inherent meaning of health and well-being is necessary to advance the competence needed by healthcare professionals (Jormfeldt et al., 2018). Profound understanding of individual experiences of health and well-being is a crucial prerequisite for health professionals to sufficiently be able to support human health and well-being in a numerous of care and support contexts in our society. Most European countries have adopted a traditional view of health mainly emphasizing objectively observed absence of symptoms of disease (Keogh et al., 2017), and the promotion of positive dimensions of health is a challenge among health care settings in our contemporary society (Jormfeldt, 2011). Accordingly, the fundamental objective of qualitative research regarding health and well-being is to contribute to an extended and deepened understanding of the contents as well as different aspects of meaning related to individual experiences of health and well-being.

Qualitative methods are to be used as guidelines (Polkinghorne, 2006) to ensure transferability and replicability in studies involving individuals’ expressions of experiences and interactions with oneself, others and the environment. There are descriptive and theory-generating qualitative methods, and it is important that the specific research question guides the choice of qualitative research method (Hallberg, 2013). Irrespective of the method used, qualitative or quantitative, no research results can claim the right to be exclusively applicable in every situation to every single unique person at any time (Hallberg, 2008). Quality of research, irrespective of method, should meet high standards of quality, validity and trustworthiness to address issues of generalizability of findings and representativeness of subjects. The result of a qualitative study should be able to transfer, generalize and reproduce to other similar groups fulfilling the same selection criteria and other relevant circumstances. It is reasonable, as Hallberg (2013) argues, that if another researcher tries to replicate a qualitative study the results should be similar if based on the same theoretical perspective, principles for sampling, types of data and procedures for analyzing data.

The advance of qualitative research is vital to the development of human knowledge and wisdom regarding health and well-being, not least considering competence among health care professionals in the numerous care and support contexts in our society. In discussions aiming to develop qualitative empirical research it is important to avoid being stuck in the description—interpretation dichotomy described by Dahlberg and Dahlberg (2019) in favour of dynamic discussions emphasizing the value of both content analyses as well as more complex analyses of meaning in qualitative studies. Academic and methodically based discussions are essential in order to advance credibility, applicability, transferability and suitability in qualitative studies and to counteract stagnation and lack of progress regarding qualitative approaches aiming to reach extended and deepened understanding of health and well-being.

## References

[CIT0001] Dahlberg H., & Dahlberg K. (2019). The question of meaning—A momentous issue for qualitative research. *International Journal of Qualitative Studies on Health and Well-being*, 14(1), 1598723. 30957670 10.1080/17482631.2019.1598723 PMC6461075

[CIT0002] Deegan P. (1996). Recovery as a journey of the heart. *Psychiatric Rehabilitation Journal*, 19(3), 91–2.

[CIT0003] Hallberg L. (2008). Some reflections on qualitative research. *International Journal of Qualitative Studies on Health and Well-being*, 3(2), 66–67.

[CIT0004] Hallberg L. (2013). Quality criteria and generalization of results from qualitative studies. *International Journal of Qualitative Studies on Health and Well-being*, 8(1), 20647. 10.3402/qhw.v8i0.20647 PMC3591510 23469988

[CIT0005] Holloway I., & Todres L. (2007). Thinking differently: Challenges in qualitative research. *International Journal of Qualitative Studies on Health and Well-being*, 2(1), 12–18.

[CIT0006] Jormfeldt H. (2011). Supporting positive dimensions of health, challenges in mental health care. *International Journal of Qualitative Studies on Health and Well-being*, 6, 7126. 10.3402/qhw.v6i2.7126 PMC3105893 21637739

[CIT0007] Jormfeldt H., Arvidsson B., Svensson B., & Hansson L. (2008). Construct validity of a health questionnaire intended to measure the subjective experience of health among patients in mental health services. *Journal of Psychiatric and Mental Health Nursing*, 15, 238–245. 18307653 10.1111/j.1365-2850.2007.01219.x

[CIT0008] Jormfeldt H., Doyle L., Heikki. E., Lahti M., Higgins A., Keogh B., … Kilkku N. (2018). Master’s level mental health nursing competencies, a prerequisite for equal health among service users in mental health care. *International Journal of Qualitative Studies on Health and Well-being*, 13, 1502013. 10.1080/17482631.2018.1502013 PMC6084491 30067476

[CIT0009] Keogh B., Doyle L., Ellilä H., Higgins A., Jormfeldt H., Lahti M., … Kilku N. (2017). Developing e-learning materials in mental health: The eMenthe Project. *Mental Health Practice*, 20(5), 36–37.

[CIT0010] Polkinghorne D. E. (2006). An agenda for the second generation of qualitative studies. *International Journal of Qualitative Studies on Health and Well-being*, 1(2), 68–77.

